# AI-derived epicardial fat measurements improve cardiovascular risk prediction from myocardial perfusion imaging

**DOI:** 10.1038/s41746-024-01020-z

**Published:** 2024-02-03

**Authors:** Robert J. H. Miller, Aakash Shanbhag, Aditya Killekar, Mark Lemley, Bryan Bednarski, Serge D. Van Kriekinge, Paul B. Kavanagh, Attila Feher, Edward J. Miller, Andrew J. Einstein, Terrence D. Ruddy, Joanna X. Liang, Valerie Builoff, Daniel S. Berman, Damini Dey, Piotr J. Slomka

**Affiliations:** 1 Departments of Medicine (Division of Artificial Intelligence in Medicine), Imaging and Biomedical Sciences Cedars-Sinai Medical Center, Los Angeles, CA USA; 2 Department of Cardiac Sciences, University of Calgary, Calgary, AB Canada; 3 Signal and Image Processing Institute, Ming Hsieh Department of Electrical and Computer Engineering, University of Southern California, Los Angeles, CA USA; 4 Section of Cardiovascular Medicine, Department of Internal Medicine, Yale University School of Medicine, New Haven, CT USA; 5 Division of Cardiology, Department of Medicine, and Department of Radiology, Columbia University Irving Medical Center and New York-Presbyterian Hospital, New York, NY USA; 6 Division of Cardiology, University of Ottawa Heart Institute, Ottawa, ON Canada

**Keywords:** Medical research, Anatomy, Risk factors

## Abstract

Epicardial adipose tissue (EAT) volume and attenuation are associated with cardiovascular risk, but manual annotation is time-consuming. We evaluated whether automated deep learning-based EAT measurements from ungated computed tomography (CT) are associated with death or myocardial infarction (MI). We included 8781 patients from 4 sites without known coronary artery disease who underwent hybrid myocardial perfusion imaging. Of those, 500 patients from one site were used for model training and validation, with the remaining patients held out for testing (*n* = 3511 internal testing, *n* = 4770 external testing). We modified an existing deep learning model to first identify the cardiac silhouette, then automatically segment EAT based on attenuation thresholds. Deep learning EAT measurements were obtained in <2 s compared to 15 min for expert annotations. There was excellent agreement between EAT attenuation (Spearman correlation 0.90 internal, 0.82 external) and volume (Spearman correlation 0.90 internal, 0.91 external) by deep learning and expert segmentation in all 3 sites (Spearman correlation 0.90–0.98). During median follow-up of 2.7 years (IQR 1.6–4.9), 565 patients experienced death or MI. Elevated EAT volume and attenuation were independently associated with an increased risk of death or MI after adjustment for relevant confounders. Deep learning can automatically measure EAT volume and attenuation from low-dose, ungated CT with excellent correlation with expert annotations, but in a fraction of the time. EAT measurements offer additional prognostic insights within the context of hybrid perfusion imaging.

## Introduction

Epicardial adipose tissue (EAT) is a potentially valuable marker of cardiovascular risk, which can be evaluated from chest computed tomography (CT). EAT is involved in bidirectional signaling with coronary arteries and myocardium, influencing both inflammation and fibrosis^1–3^. Both the volume and attenuation of EAT are associated with risk of cardiovascular events^4–6^. However, manual annotation of EAT is time consuming—taking approximately 15 min per case—and therefore has never been integrated into routine clinical practice. Deep learning can automatically measure EAT from chest CT in less than 2 s, with good agreement with manual annotation^7^. Additionally, these automated measurements have also been associated with cardiovascular events^8,9^. However, this approach has not been applied to the low-dose, ungated CT scans which are always acquired for hybrid myocardial perfusion imaging (MPI). Hybrid MPI refers to nuclear cardiology studies assessing cardiac perfusion, where the low-dose CT scans (acquired without synchronization with the cardiac cycle, also called ungated) are utilized to correct for soft-tissue attenuation artifacts.

MPI is increasingly acquired on hybrid camera systems^10^, allowing physicians to integrate functional and anatomic information when estimating cardiovascular risk. Coronary artery calcium (CAC) allows physicians to evaluate the extent of coronary atherosclerosis^11–14^, while perfusion abnormalities^15^ and ventricular function^16,17^ allow physicians to evaluate the functional significance of coronary artery disease (CAD). However, these measurements do not evaluate systemic inflammation or metabolic abnormalities which are reflected in EAT^1–3,18–20^.

Accordingly, we evaluated whether deep learning could be utilized to efficiently and automatically measure EAT on low-dose, ungated CT and whether these measurements are associated with risk of myocardial infarction (MI) or death.

## Results

### Population characteristics

**Fig. 1:**
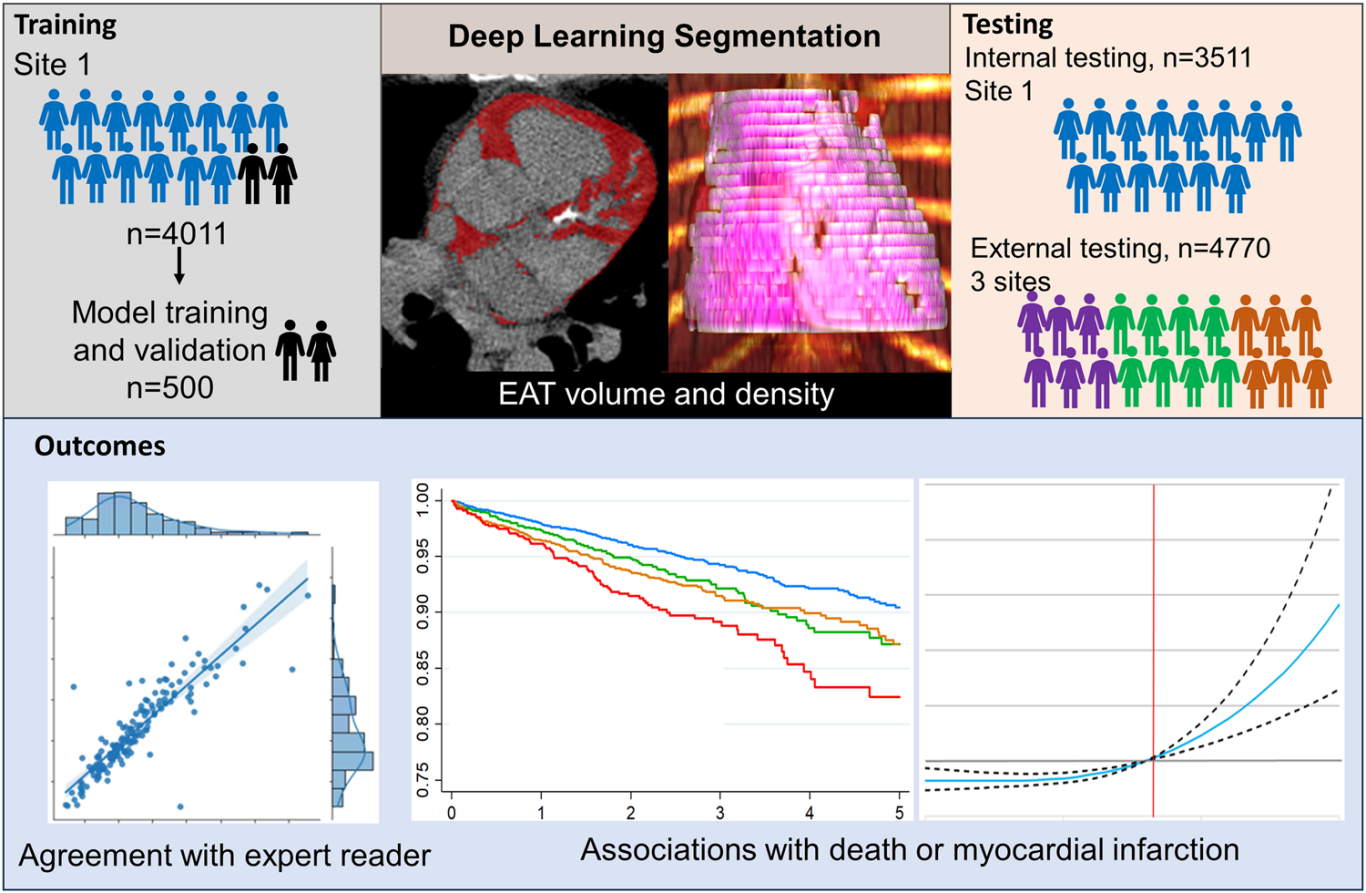
Overview of study design. The deep learning model for epicardial adipose tissue (EAT) segmentation was trained and then tested in internal and external testing populations. Outcomes included agreement with expert reader segmentation and associations with death or myocardial infarction (MI).

**Fig. 2:**
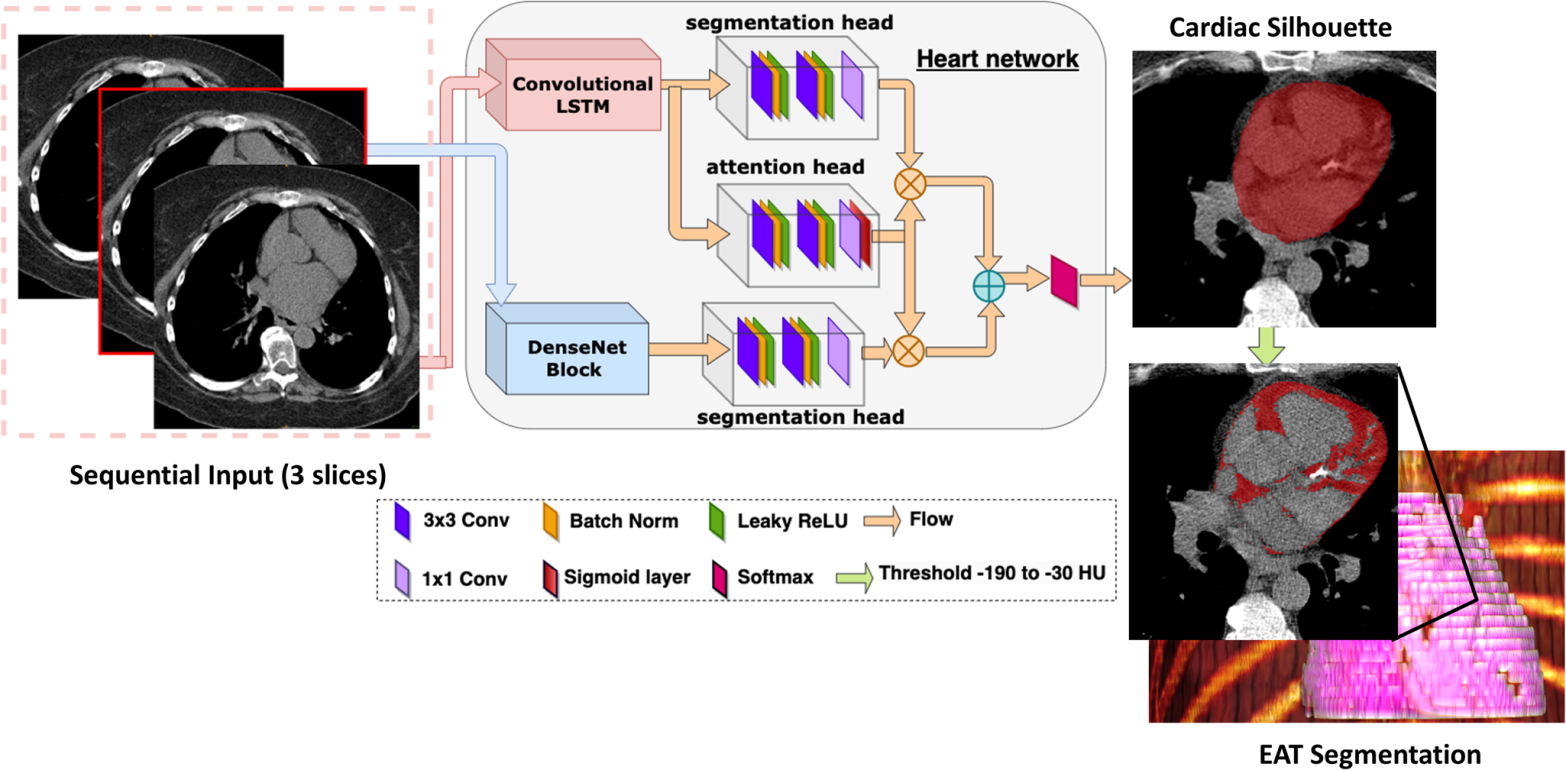
Overview of deep learning model architecture. A convolutional long short-term memory (LSTM) model is used to generate a cardiac silhouette mask. Attenuation thresholds are then applied to the mask to automatically segment epicardial adipose tissue (EAT).

Overview of the study design is shown in Fig. 1. The population was split with 500 patients from one site used for model training and validation and 8281 patients for testing (*n* = 3511 internal testing, *n* = 4770 external testing). The model architecture is shown in Fig. 2. Population characteristics for the training and internal testing patients are shown in Supplementary Table 1. Patients in the training population were older (median age 66.5 vs 63.0, *p* < 0.001) and more likely to be male (61.6% vs 50.2%, *p* < 0.001). The population characteristics for the internal and external testing populations are shown in Table 1.

**Table 1:** Patient characteristics for internal and external testing populations.

	Internal testing (*n* = 3511)	External testing (*n* = 4770)	*p*-value
Age, median (IQR)	63 (55, 72)	65 (57, 73)	<0.001
Male, *n* (%)	1764 (50.2%)	2402 (50.4%)	0.92
Body mass index, median (IQR)	29.5 (25.5, 34.2)	30.5 (26.2, 35.2)	<0.001
Hypertension, *n* (%)	2099 (59.8%)	2766 (58.0%)	0.100
Diabetes Mellitus, *n* (%)	829 (23.6%)	1314 (27.5%)	<0.001
Dyslipidemia, *n* (%)	1698 (48.4%)	1698 (43.9%)	<0.001
Family History, *n* (%)	536 (15.3%)	1512 (31.7%)	<0.001
Smoking, *n* (%)	696 (19.8%)	546 (11.4%)	<0.001
Stress TPD, median (IQR)	2.0 (0.6, 4.3)	4.2 (1.6, 8.7)	<0.001
Stress LVEF, median (IQR)	65.8 (57.9, 73.0)	64.8 (56.3 – 73.2)	0.004
DL CAC score, median (IQR)	29 (0, 382)	11 (0, 192)	<0.001
EAT volume, median (IQR)	100.9 (70.8, 145.5)	105.4 (71.7, 148.3)	0.038
High EAT volume, *n* (%)	901 (25.7%)	1291 (27.1%)	0.151
Median EAT attenuation, median (IQR)	−69 (−74, -65)	−67 (−71, −64)	<0.001
High median EAT attenuation, *n* (%)	800 (22.8%)	1335 (28.0%)	<0.001

*CAC* coronary artery calcium, *DL* deep learning, *EAT* epicardial adipose tissue, *IQR* interquartile range, *LVEF* left ventricular ejection fraction, *MI* myocardial infarction, *TPD* total perfusion deficit.

**Fig. 3:**
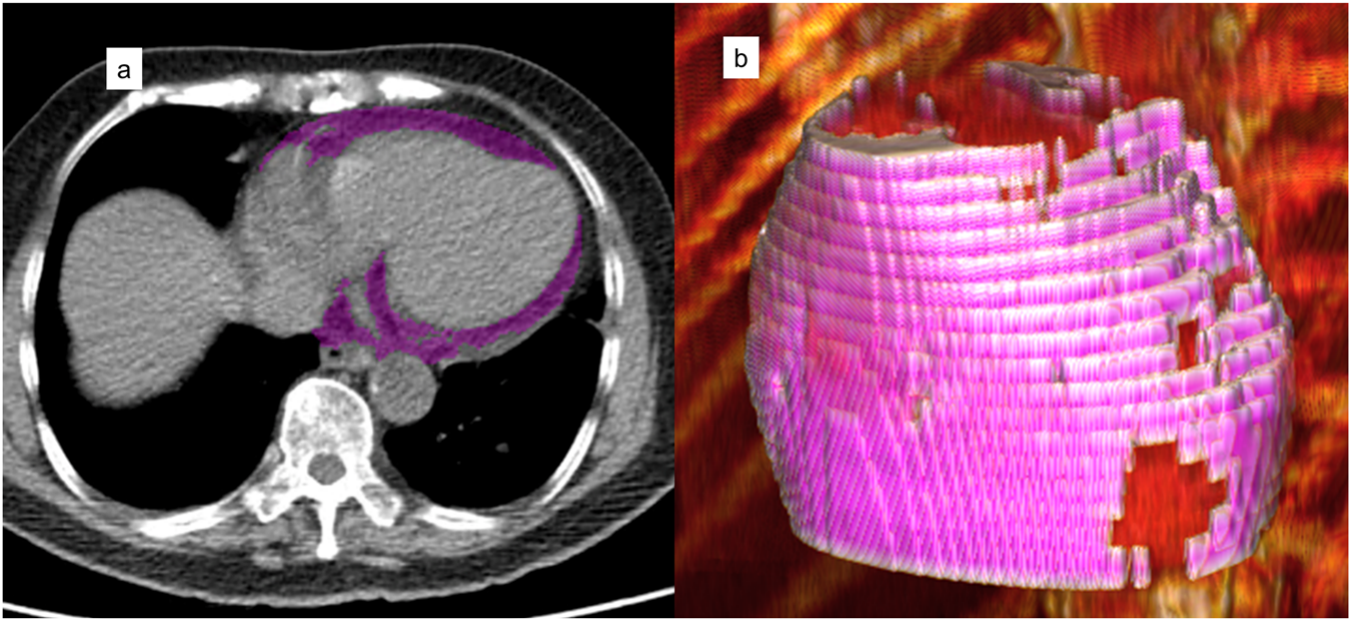
Example of EAT segmentation. The deep learning model segments epicardial adipose tissue (EAT) for each slice, shown in purple (**a**). The combined three-dimensional volume is used to quantify EAT volume (**b**). The patient was a 70-year-old woman with body mass index of 21.5 kg/m^2^, but EAT volume was elevated at 209 mL. The patient died 425 days after the scan.

In the combined internal and external testing populations, patients with BMI ≥ 30 kg/m^2^ were more likely to have high EAT volume (37.0% vs 15.7%, *p* < 0.001). However, almost a third of patients with high EAT volume (29.4%) had a BMI < 30 kg/m^2^. Example of EAT segmentation in a patient with a high EAT volume (209 mL), but low BMI (21.5 kg/m2) is shown in Fig. 3.

### Comparison of manual and DL-based EAT measurements

**Fig. 4:**
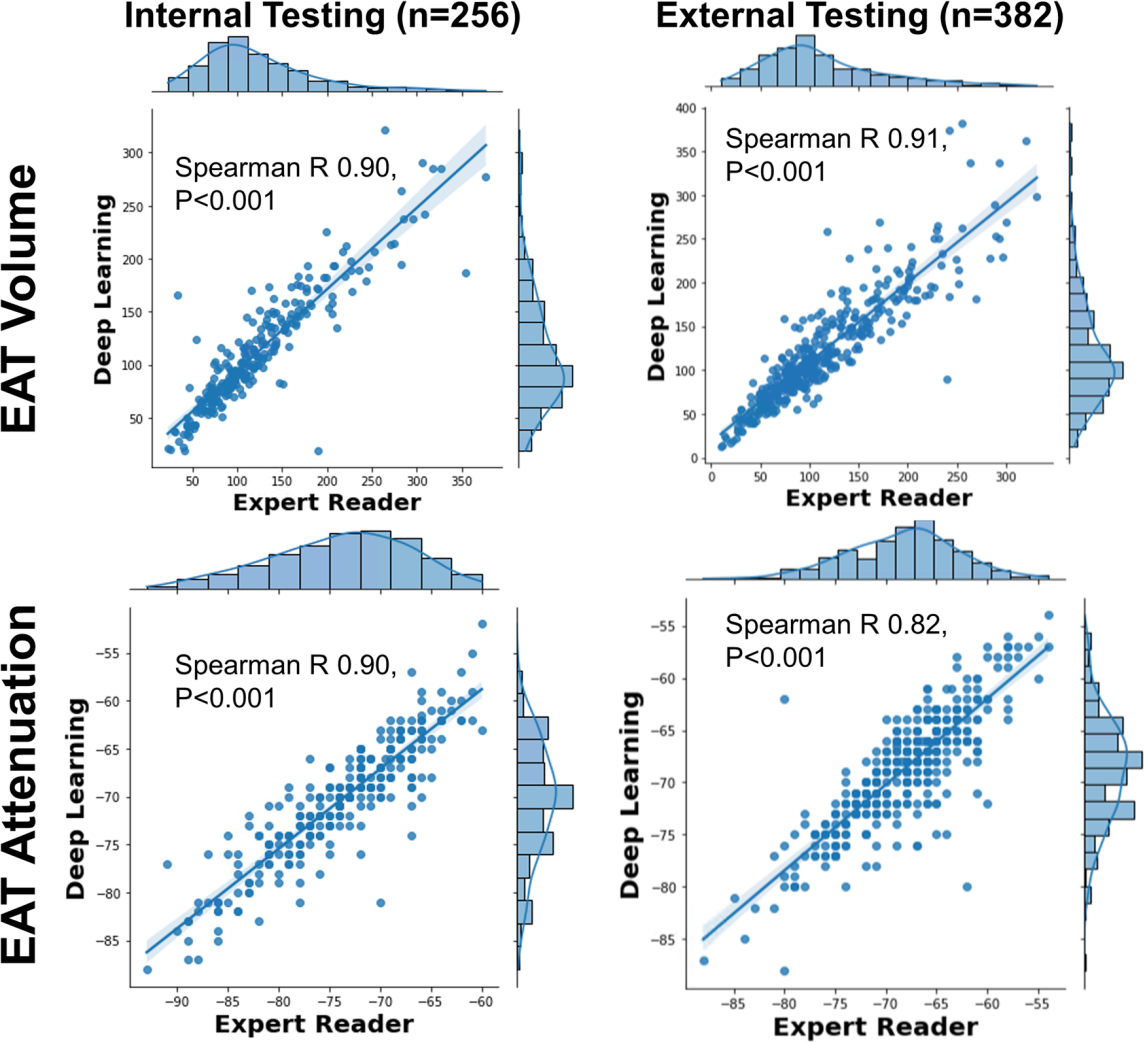
Correlation between experts and deep learning. Epicardial adipose tissue (EAT) attenuation and volume measurements are shown for deep learning and expert interpreters.

**Fig. 5:**
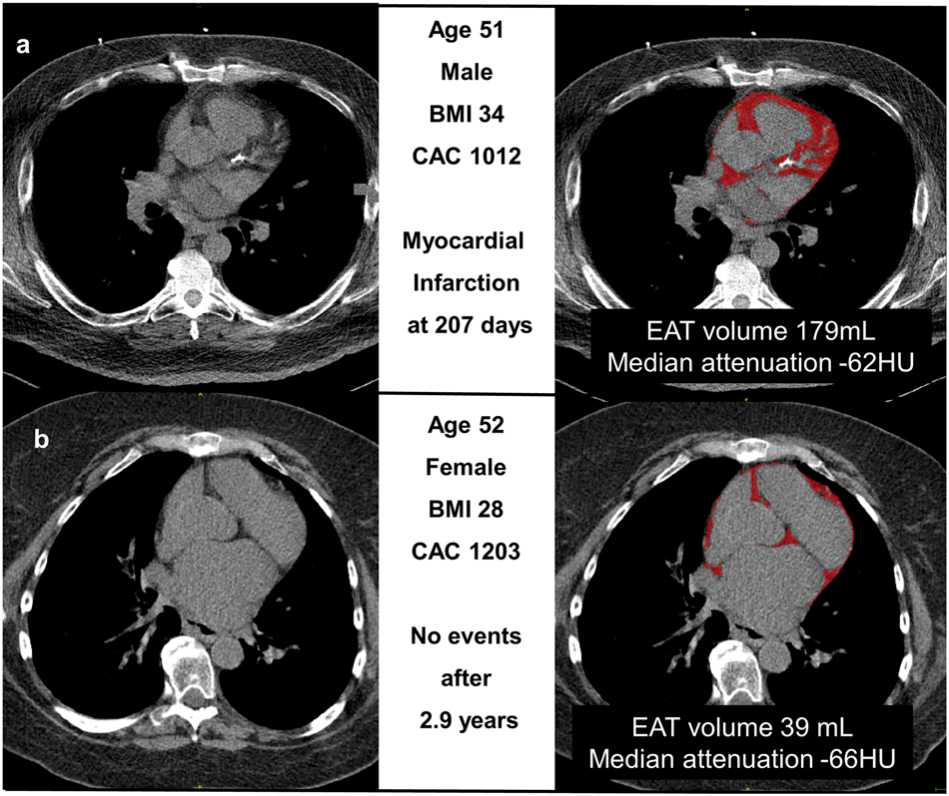
Case examples. **a** 51-year-old man with a body mass index (BMI) of 34, coronary artery calcium 1012, and normal perfusion. Epicardial adipose tissue (EAT) volume (red overlay) was 179 mL and median attenuation −62 (both high risk). He experienced a myocardial infarction 207 days after the scan. **b** 52-year-old woman with BMI 28, CAC 1203, and abnormal perfusion. Her EAT volume (red overlay) was 39 mL and median attenuation was -66 (both low risk). She was managed medically and did not experience death or myocardial infarction during 2.9 years of follow-up.

Manual EAT measurements took a mean time of 15 min. DL EAT measurements took a mean time <2 s. The per-pixel EAT attenuation values for one patient are shown in Supplementary Fig. 1. A subset of patients from the testing population underwent both manual and DL-based EAT measurements in each site (internal *n* = 256, external *n* = 382). Figure 4 shows the correlation between median EAT attenuation measurements for internal (Spearman correlation 0.90) and external testing (Spearman correlation 0.82). Similarly, the correlation for EAT volume was excellent in internal (Spearman correlation 0.90) and external testing (Spearman correlation 0.91). Bland-Altman plots (Supplementary Fig. 1) demonstrate that the agreement between DL and an expert reader was comparable to the agreement between two expert readers. Two case examples of EAT annotation by DL are shown in Fig. 5.

### Association with outcomes

During a median follow-up of 2.7 years (IQR 1.6–4.9), 565 patients experienced death or MI (first event MI in 147 patients and death in 418 patients). Population characteristics for patients who experienced death or MI compared to patients who did not are shown in Supplementary Table 2. Patients who experienced death or MI had higher EAT volume (median 108.6 vs 103.2, *p* = 0.011). Additionally, they were more likely to have median EAT attenuation >-64 HU (31.2% vs 25.4%, *p* = 0.003).

**Fig. 6:**
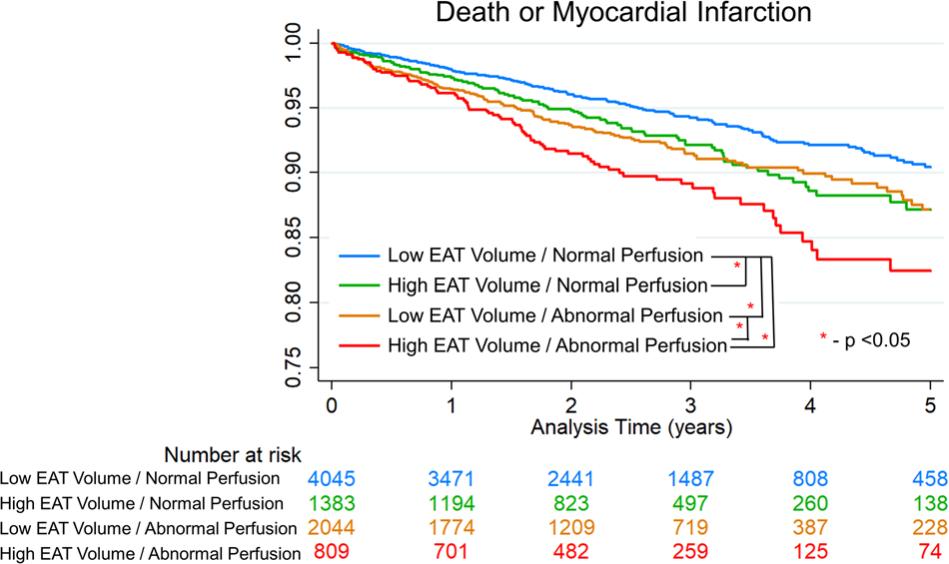
Kaplan–Meier curves stratified by epicardial adipose tissue (EAT) volume and perfusion. Analysis of the combined internal and external testing population.

The optimal cut-off for EAT volume was 144 mL. Kaplan–Meier curves stratified by EAT volume and perfusion findings are shown in Fig. 6. Compared to patients with normal EAT volume and normal perfusion, patients with abnormal EAT volume and normal perfusion were at increased risk of death or MI (unadjusted hazard ratio [HR] 1.40, 95% CI 1.11–1.77, *p* = 0.005). Patients with abnormal perfusion and abnormal EAT volume were at the highest risk (unadjusted HR 2.12, 95% CI 1.65 – 2.72, *p* < 0.001), followed by patients with abnormal perfusion and normal EAT volume (unadjusted HR 1.52, 95% CI 1.24–1.86, *p* < 0.001). Among patients with abnormal perfusion, abnormal EAT volume was associated with increased risk (unadjusted hazard ratio [HR] 1.40, 95% CI 1.08–1.82, *p* = 0.011).

The optimal cut-off for median EAT attenuation was -64 HU. Kaplan–Meier curves stratified by EAT volume and attenuation for both testing populations are shown in Supplementary Fig. 2. Compared to patients with low EAT volume and attenuation, those with elevated EAT attenuation alone (unadjusted HR 1.38, 95% CI 1.13–1.70, p = 0.002) and elevated EAT volume alone (unadjusted HR 1.55, 95% CI 1.26–1.89, *p* < 0.001) experienced higher event rates. Patients with elevated EAT volume and attenuation were at the highest risk (unadjusted HR 2.85, 95% CI 1.71–4.73, *p* < 0.001).

Kaplan-Meier survival curves for patients stratified by EAT volume are shown in Supplementary Fig. 3. Patients with EAT volume >144 mL were more likely to experience death or MI in the internal and external testing populations (log rank *p*-value < 0.05 for both). Kaplan–Meier survival curves for patients stratified by EAT attenuation are shown in Supplementary Fig. 4.

### Multivariable models

**Fig. 7:**
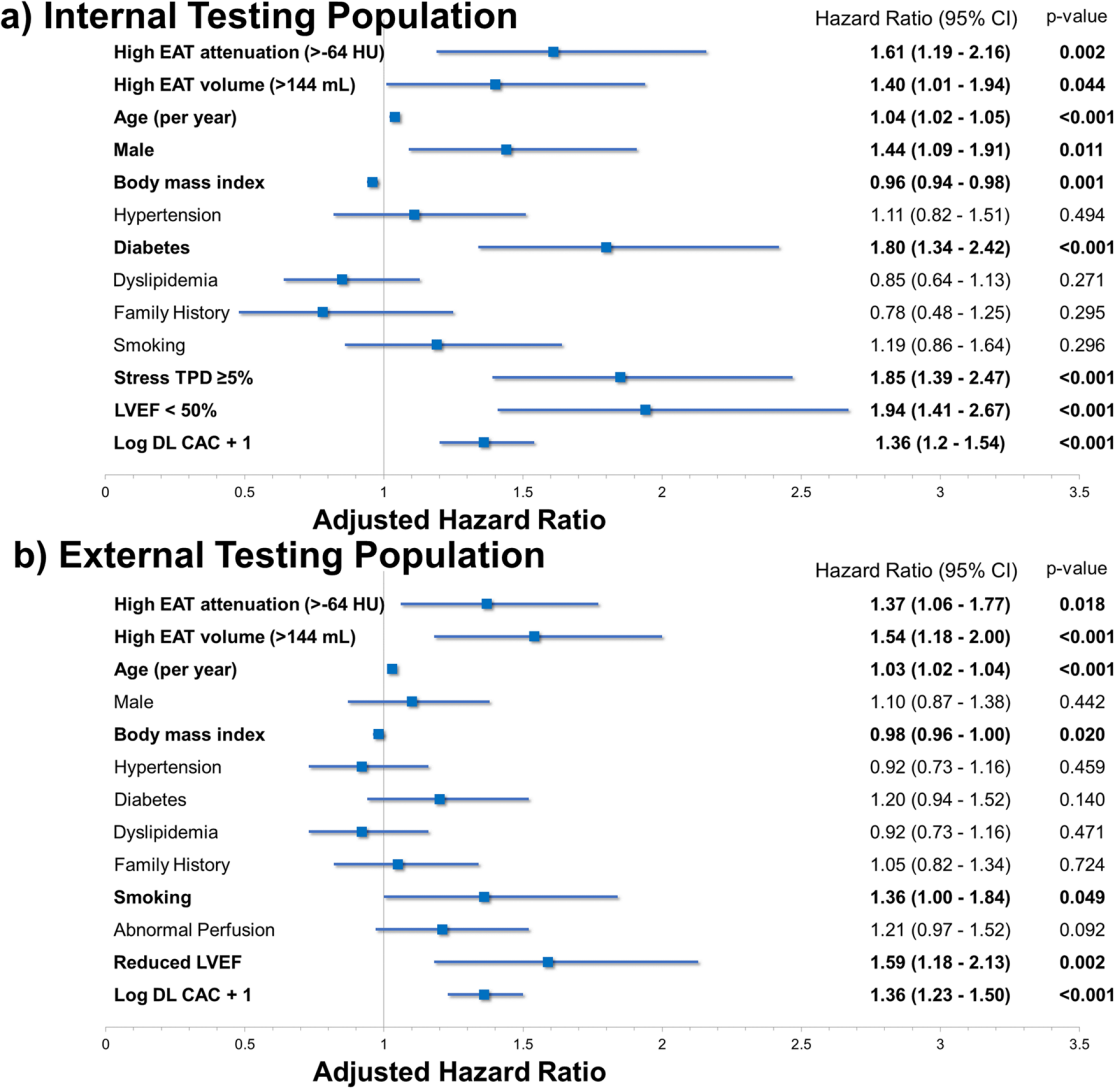
Multivariable model results. Abnormal epicardial adipose tissue (EAT) volume and attenuation were independently associated with death or myocardial infarction in the internal (**a**) and external testing populations (**b**). Variables with significant associations in bold. CAC coronary artery calcium, CI confidence interval, DL deep learning, HU Hounsfield units.

The multivariable analyses are summarized in Fig. 7. After accounting for age, sex, BMI, medical history, perfusion, LVEF, and CAC, high EAT volume was associated with an increased risk of death or MI in the internal testing population (adjusted HR 1.40, 95% CI 1.01–1.94, *p* = 0.044) and external testing population (adjusted HR 1.54, 95% CI 1.18–2.00, *p* < 0.001). Similarly, high EAT attenuation was an independent predictor of death or MI in the internal (adjusted HR 1.61, 95% CI 1.19–2.16, *p* = 0.002) and external testing populations (adjusted HR 1.37, 95% CI 1.06–1.77, *p* = 0.018). In external testing, there was no significant difference in risk by sex for EAT volume (interaction *p*-value = 0.907) or EAT attenuation (interaction *p*-value 0.668).

**Fig. 8:**
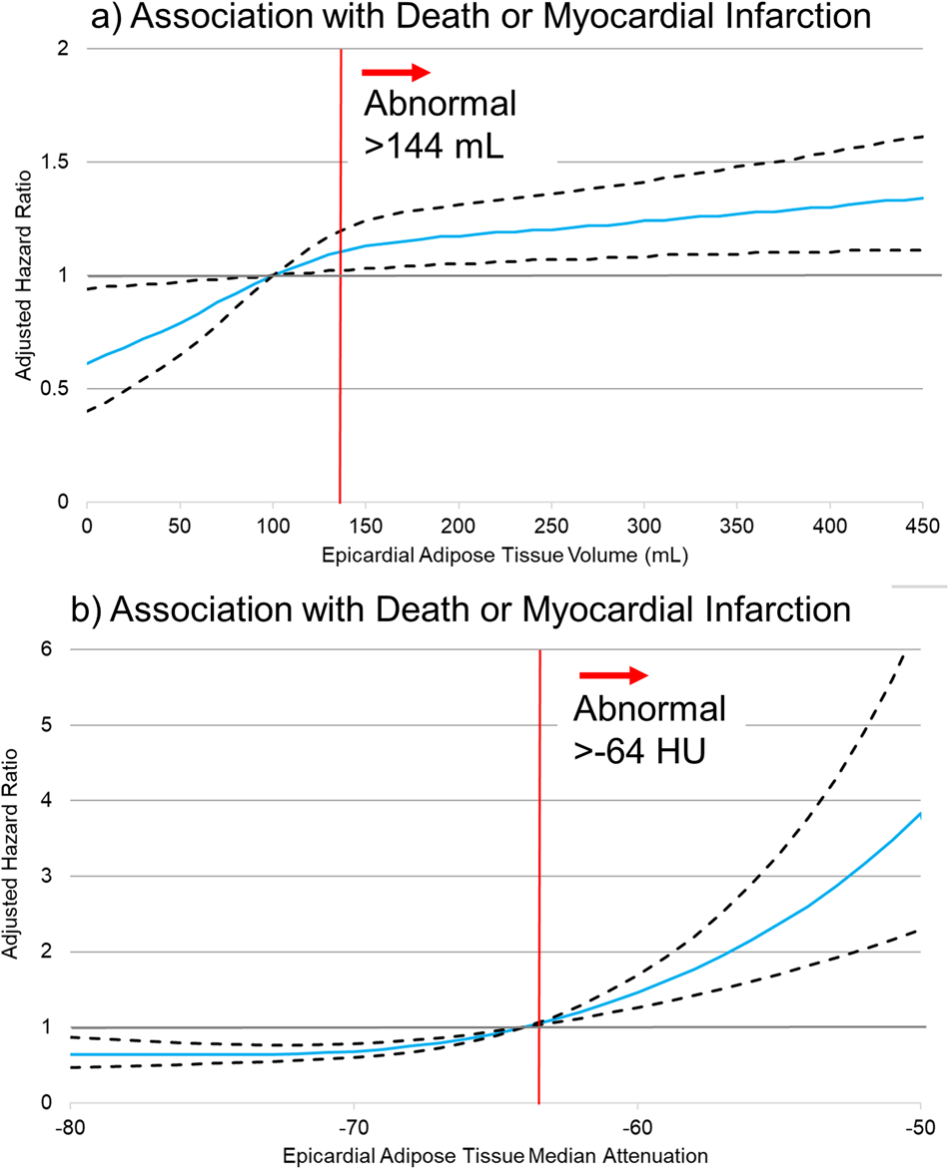
Non-Linear Associations. Adjusted associations between epicardial adipose tissue (EAT) volume (**a**) and attenuation (**b**) with death or myocardial infarction. Adjusted hazard ratios (blue lines) and 95% confidence intervals (black dashed lines). Thresholds for abnormal values are shown as red lines. Model adjusted for the same variables outlined in Fig. 6, but with EAT measures modeled as non-linear continuous variables.

Adjusted associations between EAT attenuation and volume, modeled as non-linear continuous variables, are shown in Fig. 8. Both populations were included since no specific threshold for EAT volume or attenuation were applied. Higher EAT volume was associated with increased risk, but there was a plateau above 150 mL. Higher EAT attenuation was also associated with increased risk; however, the increase was exponential for values above −60 HU.

## Discussion

We evaluated a DL model which measures EAT attenuation and volume from low-dose, ungated CT. We showed that the DL model had an excellent correlation with expert segmentation, demonstrating similar agreement as was seen between two experts, but in a fraction of the time. We went on to demonstrate that patients with higher EAT volume or higher EAT attenuation were more likely to experience death or MI during follow-up. Importantly, we also demonstrated that the increase in risk was independent of other imaging findings including perfusion, LVEF and CAC in large internal and external testing populations.

EAT has primarily been quantified from ECG-gated cardiac CT. The gating minimizes cardiac motion, simplifying the task of segmenting EAT. EAT thickness (measured by echocardiography) varies through the cardiac cycle^21^, with some authors suggesting that measurements should be averaged across three points in the cardiac cycle^22^. Grodecki et al. demonstrated that DL could quantify EAT from ungated chest CT images in patients with COVID-19 infection^23^. Similar to our results, they found that both higher EAT volume and attenuation were associated with increased risk. In the present work, we demonstrate that EAT can be automatically segmented by DL from ungated CT for hybrid MPI, which are acquired with lower radiation doses and subsequently lower image quality compared to standard ungated chest CT. The DL EAT measurements had an excellent correlation with expert EAT annotations for both volume and median attenuation. However, the DL annotations were automatically generated within a few seconds compared to approximately 15 minutes for expert annotations. This rapid and accurate quantification can facilitate routine evaluation within a typical clinical workflow. We also expand on existing studies by demonstrating that EAT volume and attenuation carry prognostic significance in patients undergoing MPI. Importantly, this allowed us to evaluate the independent association of EAT with death or MI after accounting for age, sex, and medical history as well as imaging parameters including myocardial perfusion, ventricular function, and CAC.

The independent prognostic utility of EAT attenuation was expected based on the hypothesized relationships between EAT and CAD. EAT interacts with the heart through local paracrine effects and inflammatory signaling^3^. Higher EAT attenuation values may be a marker of coronary inflammation^24^. In fact, peri-coronary adipose tissue (which is a component of EAT) attenuation is positively correlated with sodium fluoride uptake^25^, which is a measure of microcalcification that reflects atherosclerotic plaque activity^26^. Additionally, while CAD may lead to inflammatory changes within EAT, EAT inflammation also accelerates atherosclerosis in adjacent coronary arteries^27^. Therefore, measurement of EAT attenuation allows physicians to capture relevant information regarding cardiac inflammatory state which previously could not be evaluated with MPI.

We also anticipated that higher EAT volume would be associated with increased cardiovascular risk. Prior studies have shown that visceral fat is superior to BMI and waist circumference for predicting cardiovascular risk^28^. EAT volume is correlated with BMI and waist circumference;^28^ however, EAT volume is a direct measure of visceral adipose tissue. In fact, in an analysis of the multi-ethnic study of atherosclerosis, EAT volume, but not BMI, was predictive of future coronary atherosclerosis^28^. In our study, while patients with elevated BMI were more likely to have high EAT volume, we found that almost one-third of patients with elevated EAT volume had a BMI < 30 kg/m2. Lastly, it is worth noting that a small study have suggested that dapagliflozin may decrease EAT volume^29^, suggesting a possible therapeutic intervention for patients with elevated EAT volume. Therefore, the measurement of EAT volume potentially provides physicians with prognostic and therapeutic information to consider following MPI.

Our study has a few important limitations in addition to its retrospective nature. We only utilized ungated CT scans and therefore EAT values may differ from those obtained from gated chest CT. However, this is representative of the CT scans typically used for attenuation correction. We used set thresholds for EAT segmentation. It is possible that the thresholds should vary according to acquisition protocol. Different thresholds would influence both EAT volume and median attenuation values. As a result, optimal cutoffs for EAT volume and attenuation may vary by protocol and may vary by sex. However, in our study the thresholds for abnormal cutoffs were established within the internal testing site, then applied to the external site which has a different acquisition protocol, suggesting that the thresholds are still generalizable. The model was trained in a random selection of patients with and without known CAD. However, patients with known CAD were excluded from the internal and external testing populations (since this can influence the association between MPI findings and outcomes). Future studies should evaluate the prognostic of EAT measurements in these patients. Additionally, cardiovascular mortality was not available in the registry, but existing methods for identifying cardiovascular mortality have limited accuracy^30^. However, we would expect even stronger associations if we were able to ascertain cardiovascular mortality since EAT is pathophysiologically related to cardiovascular outcomes. Similarly, we do not have information regarding inflammatory markers or use of anti-inflammatory medications, so we are not able to evaluate these potential pathophysiologic links. Lastly, we do not have information regarding race or ethnicity in our cohort.

Our study demonstrates that deep learning can automatically measure EAT volume and attenuation from low-dose, ungated CT with excellent correlation with expert annotations, but in a fraction of the time. EAT measurements provide independent prognostic information regarding inflammation and metabolism which complement the functional and anatomic information available through hybrid perfusion imaging.

## Methods

### Study population

The overall study design is outlined in the central illustration. This study included 8781 patients from four sites who underwent clinically indicated SPECT MPI with CTAC between 2014 and 2021. Of those, 500 patients from one center (Yale University) were utilized for model training and validation. The remaining cohort (*n* = 8281) comprised consecutive patients without known CAD (defined as previous MI or coronary revascularization)^28^ who were held out for model testing (*n* = 3511 internal testing, *n* = 4770 external testing). Patients with known CAD were excluded since this can alter the association between MPI findings and clinical outcomes^31^. The study protocol complied with the Declaration of Helsinki and was approved by the institutional review boards at each participating institution. The overall study was approved by the institutional review board at Cedars-Sinai Medical Center. Sites either obtained written informed consent or waiver of consent for the use of the de-identified data. To the extent allowed by data sharing agreements and institutional review board protocols, the data and code from this manuscript will be shared upon written request.

### Clinical data

Demographic information included: age, gender, body mass index (BMI), family history of coronary artery disease (CAD), smoking status, history of previous myocardial infarction (MI), previous revascularization, hypertension, diabetes, and dyslipidemia.

### Outcomes

The primary clinical outcome was death or MI. MI was defined as hospital admission for recent onset or worsening chest pain with elevated cardiac enzyme levels and ischemic ECG changes^32,33^, with all events adjudicated by experienced physicians at each site.

### Myocardial perfusion image analysis

Stress perfusion images were analyzed by Quantitative Perfusion SPECT (QPS) software (Cedars-Sinai Medical Center, Los Angeles, CA) without knowledge of clinical data as previously described to quantify total perfusion deficit (TPD)^34^. Abnormal myocardial perfusion was defined as stress TPD ≥ 5%. Left ventricular ejection fraction (LVEF), was calculated from post-stress gated imaging, with values < 50% considered abnormal.

### CTAC image acquisition and annotation

At Columbia University, CTAC scans were performed free breathing without ECG gating, using a helical acquisition with pitch 0.94, collimation 16 × 1.5 mm, scan length 14 mm, tube voltage 120 kVp, and effective tube current 30 mAs. At Ottawa Heart Institute, a slow-rotation CT was acquired at 120 kVp and effective tube current 20 mA s. At University of Calgary, CTAC imaging was acquired with end-expiratory breath hold with no ECG-gating, in helical mode with a slice thickness of 5 mm, tube voltage of 120 kVp and effective tube current of 16 mAs. At Yale University, CTAC images were acquired with end-expiratory breath hold with ECG-gating, in helical mode with a slice thickness of 1 mm, tube voltage of 120 kVp and effective tube current 16 mA s.

EAT was annotated for a subset of patients in both sites to allow for a comparison with expert annotations, including 100 cases which were annotated by two readers. Trained clinicians manually adjusted contours to segment EAT using a dedicated software package (QFAT; Cedars-Sinai Medical Center, Los Angeles). After segmentation, median filtering with a radius of 3 voxels was applied to limit image noise. Then thresholds were applied (−190 Hounsfield Units [HU] to −30 HU) to determine EAT volume and median EAT attenuation. Annotations were not manually performed for all cases due to the substantial amount of time required to annotate each case.

### Deep learning model

We utilized our previously validated DL model for CAC segmentation^35,36^. In brief, the system consists of two networks, the first of which is trained for segmentation of the heart silhouette and the second network was trained to segment CAC. CAC scores are automatically obtained from the DL segmentations using established methods^37^.

The CAC model was modified to allow simultaneous quantification of EAT volume and attenuation. The heart silhouette is utilized to identify all tissue adjacent to the heart, but inside the pericardium. After segmentation, median filtering with a radius of 3 voxels was applied to limit image noise—the same process that was applied to manually segmented images. Thresholds for attenuation (−190 HU to −30 HU)^23^ are then applied to segment EAT within the heart silhouette. Once the EAT segmentation is completed, EAT volume and attenuation are calculated using a dedicated software package (QFAT; Cedars-Sinai Medical Center, Los Angeles). The distribution of EAT attenuation values is skewed so we evaluated per-patient median attenuation. An example of EAT segmentation for one patient is shown in Supplementary Fig. 5.

### Statistical analysis

Continuous variables were summarized as mean (standard deviation [SD]) if normally distributed and compared using a Student’s t-test. Continuous variables that were not normally distributed were summarized as median (interquartile range [IQR]) and compared using a Mann-Whitney U-test. Correlations between continuous variables were assessed using Spearman’s rank correlation coefficient. Since the optimal cutoff for EAT volume and attenuation in CT attenuation correction imaging is unknown, we used the Youden index to identify optimal cutoffs in the internal testing population. Associations with death or MI were assessed with univariable and multivariable Cox proportional hazards analyses. The multivariable model included age, sex, medical history, stress TPD and stress LVEF. We evaluated for sex-based differences by evaluating interactions between sex and EAT volume and EAT attenuation in the multivariable model. We also evaluated associations between death or MI with EAT volume and attenuation modeled as continuous variables. In this analysis, EAT volume and attenuation were modeled as cubic splines with 3 knots.

All statistical tests were two-sided, and a *p*-value < 0.05 was considered statistically significant. All analyses were performed using Stata/IC version 13.1 (StataCorp, College Station, Texas, USA) and R (version 4.1.2).

### Supplementary information

Supplemental Material

## Data Availability

To the extent allowed by data sharing agreements and IRB protocols, the data from this manuscript will be shared upon written request.

## Code availability

The underlying code for this study is not publicly available but may be made available to qualified researchers on reasonable request from the corresponding author.
