# Supplementary material for: AI-derived epicardial fat measurements improve cardiovascular risk prediction from myocardial perfusion imaging
Source: NPJ Digit Med. 2024 Feb 3;7:24. doi: 10.1038/s41746-024-01020-z (PMC10838293; doi:10.1038/s41746-024-01020-z)
Supplement: Supplementary file 1 — Supplemental Material [file 41746_2024_1020_MOESM1_ESM.pdf]

SUPPLEMENTAL MATERIAL

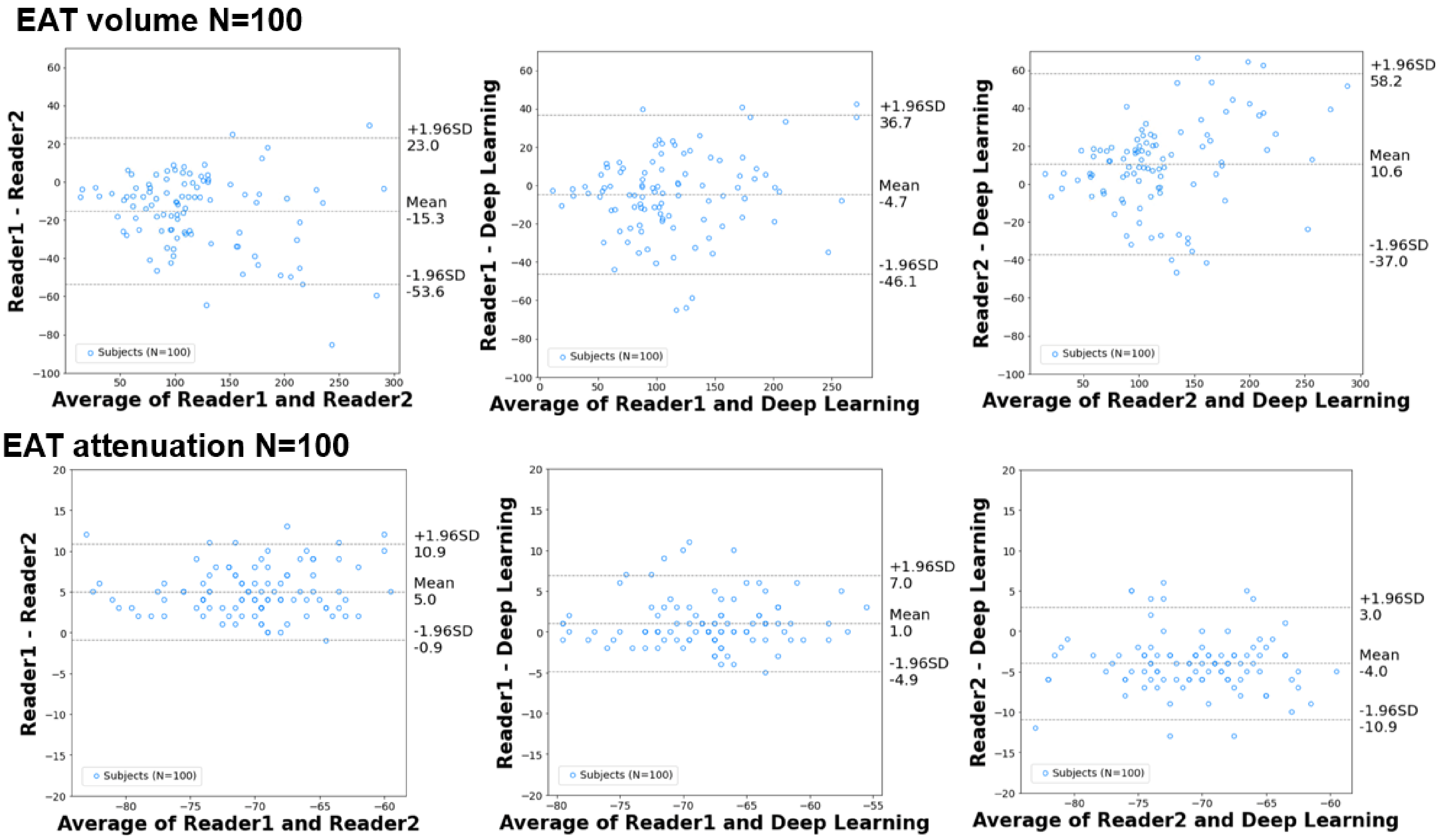

Supplemental Figure 1: Bland-Altman plots comparing measurements of epicardial adipose tissue volume and attenuation between two expert readers and deep learning. The mean bias for EAT volume for deep learning was intermediate between the two readers, with similar limits of agreement. Similar results were seen for median EAT attenuation. 1.96 standard deviation (SD) implies the 95% confidence intervals.

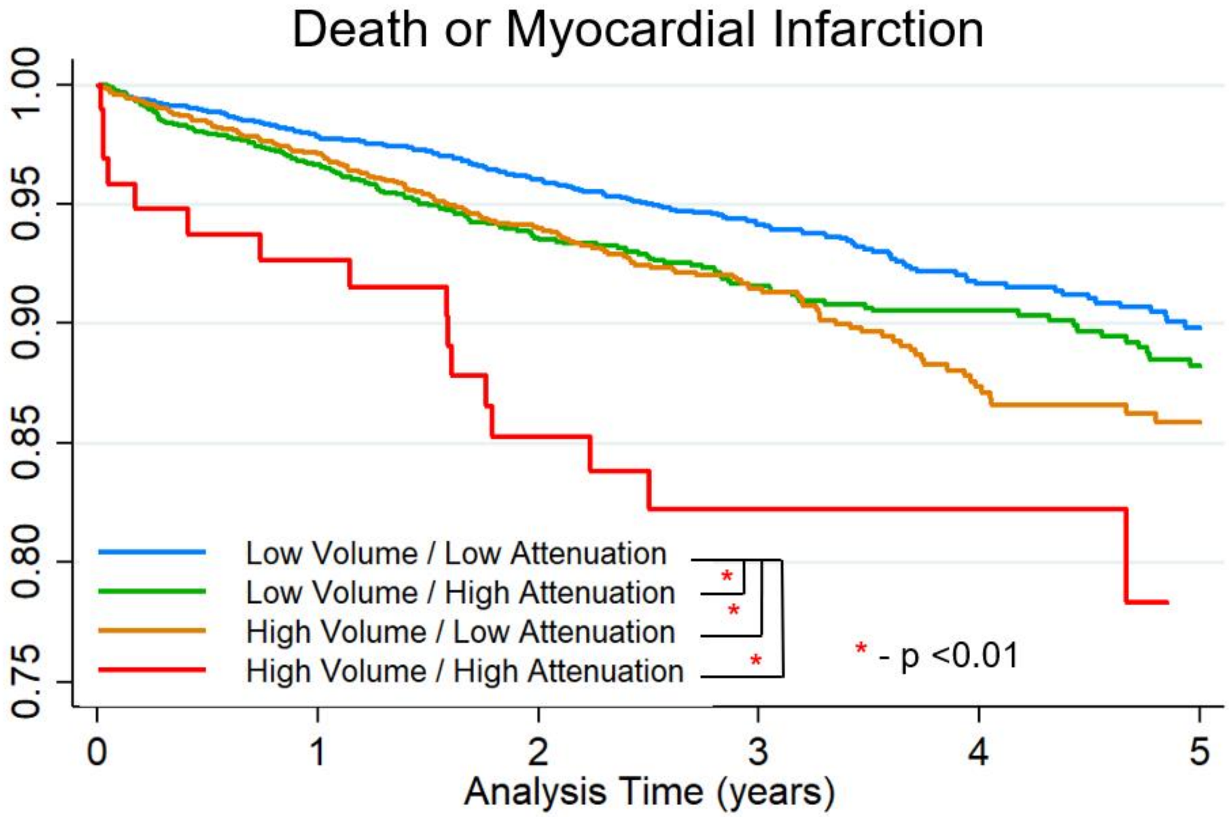

Number at risk

Low Volume/ Low Attenuation	4052	3507	2442	1422	697	384
Low Volume / High Attenuation	2037	1738	1208	784	498	302
High Volume / Low Attenuation	2094	1810	1241	717	359	200
High Volume / High Attenuation	98	85	64	39	26	12

Supplemental Figure 2: Kaplan-Meier curves for death or myocardial infarction stratified by epicardial adipose tissue (EAT) volume and attenuation in the combined internal and external testing population.

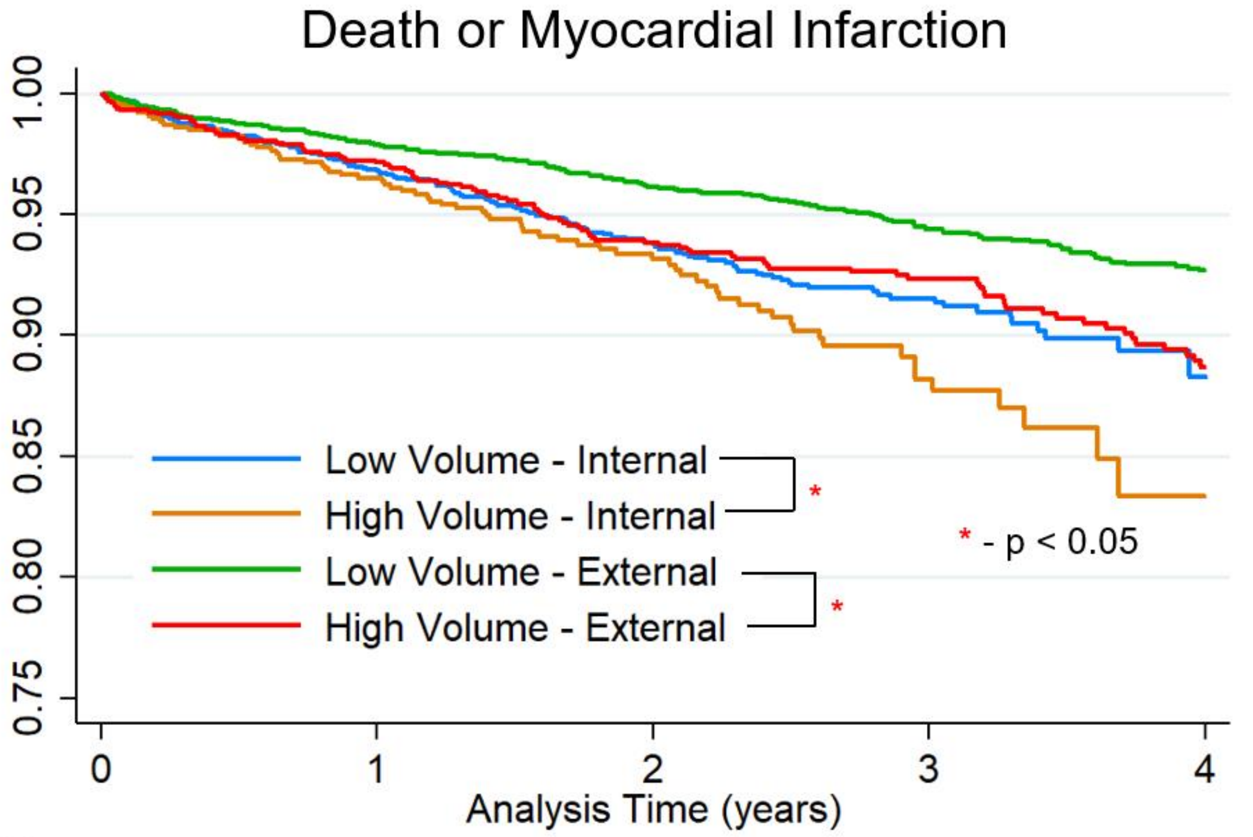

Number at risk					
Low EAT Volume - Internal	2610	2058	1250	543	64
High EAT Volume - Internal	901	716	438	180	18
Low EAT Volume - External	3479	3187	2400	1663	1131
High EAT Volume - External	1291	1179	867	576	367

Supplemental Figure 3: Kaplan-Meier curves for death or myocardial infarction stratified by epicardial adipose tissue (EAT) volume. The threshold for elevated EAT volume (>144 mL) was established in the internal testing population.

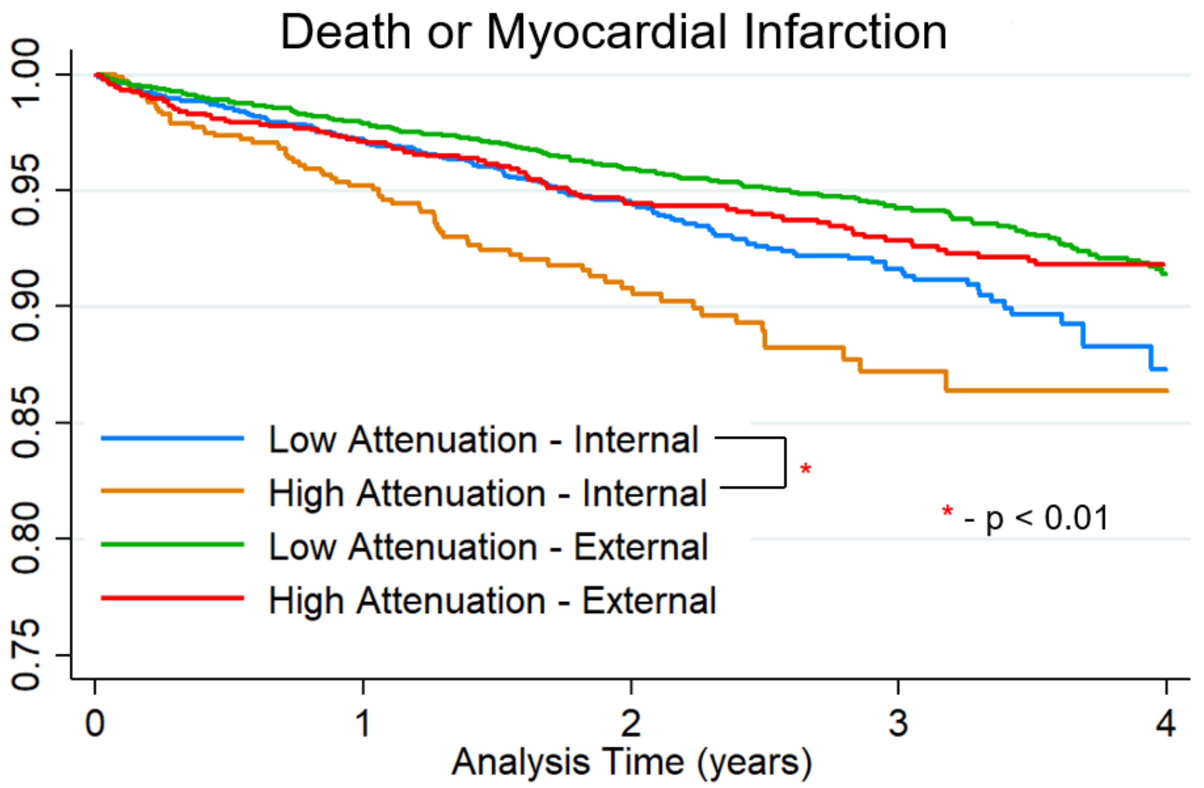

Number at risk

Low Attenuation - Internal	2711	2158	1344	587	65
High Attenuation - Internal	800	616	344	136	17
Low Attenuation - External	3435	3159	2339	1552	991
High Attenuation - External	1335	1207	928	687	507

Supplemental Figure 4: Kaplan-Meier curves for death or myocardial infarction stratified by epicardial adipose tissue (EAT) attenuation. The threshold for elevated EAT attenuation (> -64 Hounsfield Units) was established in the internal testing population.

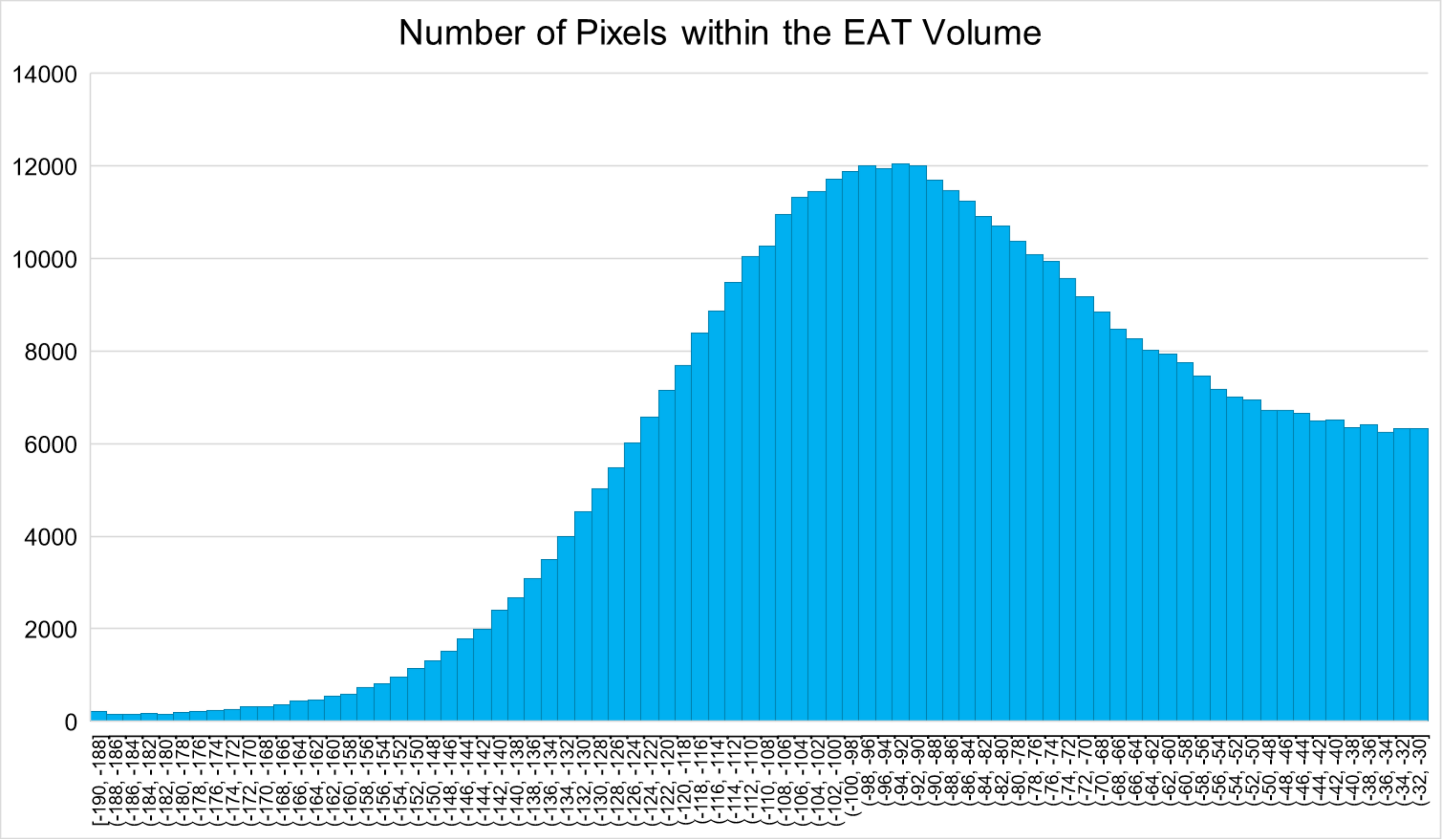

Supplemental Figure 5: Epicardial adipose tissue (EAT) attenuation values for all pixels in a 50-year-old woman with a body mass index of 30.3. The total EAT volume was 130 mL and the median EAT attenuation was -88 Hounsfield Units.

Factor	Training Population (n=500)	Internal Testing Population (n=3511)	p-value
Age, median (IQR)	66.5 (58, 74)	63 (55, 72)	<0.001
Male, n (%)	308 (61.6%)	1764 (50.2%)	<0.001
Body mass index, median (IQR)	28.6 (25.5 – 33.1)	29.5 (25.5 – 34.2)	0.042
Hypertension, n (%)	349 (70.1%)	2099 (59.8%)	<0.001
Diabetes Mellitus, n (%)	146 (29.3%)	829 (23.6%)	0.005
Dyslipidemia, n (%)	297 (59.6%)	1698 (48.4%)	<0.001
Family History, n (%)	66 (13.3%)	536 (15.3%)	0.240
Smoking, n (%)	98 (19.7%)	696 (19.8%)	0.940
Race			0.047
White	355 (71.4%)	2213 (63.1%)	
Black or African American	91 (18.3%)	772 (22.0%)	
American Indian or Alaska Native	3 (0.6%)	11 (0.3%)	
Asian	8 (1.6%)	99 (2.8%)	
Native Hawaiian or Pacific Islander	0 (0.0%)	9 (0.3%)	
Other Race / Declined to Disclose	43 (8.6%)	407 (11.6%)	
EAT Volume, median (IQR)	106.7 (76.1, 151.4)	100.9 (70.8, 145.5)	0.041
EAT Attenuation, median (IQR)	-70 (-75, -65)	-69 (-74, -65)	0.150

Supplemental Table 1: Characteristics of the training and testing populations. EAT – epicardial adipose tissue, IQR – interquartile range.

	No Death or MI (n=7716)	Death or MI (n=565)	p-value
Age, median (IQR)	64 (56, 72)	70 (61, 77)	<0.001
Male, n (%)	3834 (49.7%)	332 (58.8%)	<0.001
BMI, median (IQR)	30.2 (26, 34.9)	28.4 (24.4, 33.5)	<0.001
Hypertension, n (%)	4518 (58.6%)	347 (61.4%)	0.184
Diabetes Mellitus, n (%)	1951 (25.3%)	192 (34.0%)	<0.001
Dyslipidemia, n (%)	3532 (458%)	261 (46.2%)	0.861
Family History, n (%)	1936 (25.0%)	112 (19.8%)	0.005
Smoking, n (%)	1142 (14.8%)	100 (17.7%)	0.067
Stress TPD, median (IQR)	3.0 (1.0, 6.7)	4.0 (1.5, 8.6)	<0.001
Stress LVEF, median (IQR)	65.5 (57.4, 73.3)	60.1 (48.6, 68.9)	<0.001
DL CAC score, median (IQR)	13 (0,230)	176 (11, 966)	<0.001
EAT volume, median (IQR)	103.2 (71.2, 146.3)	108.6 (74.3, 161.9)	0.011
High EAT volume, n (%)	2004 (26.0%)	188 (33.3%)	<0.001
Median EAT attenuation, median (IQR)	-69 (-73, -64)	-68 (-72, -63)	0.045
High median EAT attenuation, n (%)	1959 (25.4%)	176 (31.2%)	0.003

Supplemental Table 2. Patient characteristics in patients who experienced major adverse cardiovascular events (MACE) compared to those who did not. BMI – body mass index, CAC – coronary artery calcium, DL – deep learning, EAT – epicardial adipose tissue, IQR – interquartile range, LVEF – left ventricular ejection fraction, MI – myocardial infarction, TPD – total perfusion deficit.
